# Research on the Physio-Biochemical Mechanism of Non-Thermal Plasma-Regulated Seed Germination and Early Seedling Development in *Arabidopsis*

**DOI:** 10.3389/fpls.2019.01322

**Published:** 2019-11-08

**Authors:** Dongjie Cui, Yue Yin, Jiaqi Wang, Zhiwei Wang, Hongbin Ding, Ruonan Ma, Zhen Jiao

**Affiliations:** ^1^Henan Key Laboratory of Ion-Beam Bioengineering, College of Agricultural, Zhengzhou University, Zhengzhou, China; ^2^School of Physics, Key Laboratory of Materials Modification by Laser, Ion and Electron Beams, Ministry of Education, Dalian University of Technology, Dalian, China

**Keywords:** non-thermal plasma, *Arabidopsis thaliana*, reactive oxygen/nitrogen species, seed germination, seedling growth, physio-biochemical mechanism

## Abstract

Non-thermal plasma holds great potentials as an efficient, economical, and eco-friendly seed pretreatment method for improving the seed germination and seedling growth, but the mechanisms are still unclear. Therefore, a plant model organism *Arabidopsis thaliana* was used to investigate the physio-biochemical responses of seeds to non-thermal plasma at different treatment times by measuring the plant growth parameters, redox-related parameters, calcium (Ca^2+^) level and physicochemical modification of seed surface. The results showed that short-time plasma treatment (0.5, 1, and 3 min) promoted seed germination and seedling growth, whereas long-time plasma treatment (5 and 10 min) exhibited inhibitory effects. The level of superoxide anion (O_2_^•−^) and nitric oxide (NO) and the intensity of infrared absorption of the hydroxyl group were significantly higher in short-time plasma treated *Arabidopsis* seeds, and the level of hydrogen peroxide (H_2_O_2_) was remarkably increased in long-time plasma treated seeds, indicating that O_2_^•−^, ·OH, and NO induced by plasma may contribute to breaking seed dormancy and advancing seed germination in *Arabidopsis*, while plasma-induced H_2_O_2_ may inhibit the seed germination. The intensity of hydroxyl group and the contents of H_2_O_2_, malondialdehyde, and Ca^2+^ in *Arabidopsis* seedlings were obviously increased with the plasma treatment time. Catalase, superoxide dismutase, and peroxidase activities as well as proline level in short-time treated seedlings were apparently higher than in control. The etching effects of plasma on seed surface were dose-dependent, spanning from slight shrinkages to detached epidermis, which also significantly increased the oxidation degree of seed surface. Therefore, the improved activities of antioxidant systems, moderate ·OH, H_2_O_2_, and Ca^2+^ accumulation and seed surface modification induced by plasma all contribute to the enhanced seedling growth of *Arabidopsis* after short-time plasma treatment.

## Introduction

In the past few decades, atmospheric non-thermal plasma has shown promising applications in the biomedical field, thus leading to the emergence of “plasma medicine” (Fridman et al., 2010). Nowadays, the agricultural applications of non-thermal plasma for decontaminating seeds, improving seed quality and crop yields as well as curing plant disease have attracted much attention due to the fact that food shortage is becoming one of the most serious global problems in this century because of the continuously growing population and decreasing arable land (Koga et al., 2015). Numerous works have shown that non-thermal plasma is a fast, uniform, economic, effective, and eco-friendly approach for stimulating seed germination and seedling growth compared with the conventional seed pretreatment methods, e.g. ultraviolet and gamma radiation, scarification, hot water soaking, and chemical reagent treatment (Li et al., 2014; Randeniya and De Groot, 2015; Mildaziene et al., 2018; Štěpánová et al., 2018). In the last years many authors studied the stimulation of the seed germination and the plant growth by using plasma generator (Živković et al., 2004; Šerá et al., 2010; Dobrin et al., 2015; Bafoil et al., 2018). Some studies have found that plasma treatment can increase seed activity including earlier germination, higher germination rate, faster growth, and other growth parameters (Šerá et al., 2010; Jiang et al., 2014), enzyme activity (Henselová et al., 2012; Surowsky et al., 2013), and the yield of plants (Yin et al., 2005). However, the detailed mechanisms underlying the stimulatory effects of plasma on seeds are still not fully understood.

Among the various constituents in plasma, reactive oxygen species (ROS) [e.g., hydroxyl radical (OH), superoxide anion (O_2_^•−^), hydrogen peroxide (H_2_O_2_), and singlet oxygen (^1^O_2_)] and reactive nitrogen species (RNS) [*e.g.*, nitric oxide (NO), nitrite (NO_2_^¯^), nitrite (NO_3_^¯^) and peroxynitrite (OONO^¯^)] are considered as the major agents for plasma-induced biological effects (Iseni et al., 2016). The ROS and RNS in plants can play dual roles, beneficial or harmful, depending on the amount (Panngom et al., 2018), which activates a variety of physiological and metabolic behaviors (such as breaking dormancy, accelerating germination, and enhancing antioxidant capacity) at low doses, while causes oxidative stress in seeds at high doses (Romero-Puertas et al., 2019). Plants are well equipped with an intrinsic antioxidant defense system comprising enzymatic antioxidants, such as superoxide dismutase (SOD), catalase (CAT), and peroxidase (POD), as well as non-enzymatic antioxidants, for instance, ascorbate, glutathione, and proline to resist the oxidative stress (Liu et al., 2018). These antioxidants are essential for stimulating physiological and developmental processes and resisting stresses (Liu et al., 2007). On the other hand, plasma-generated reactive species could also directly etch the seed husk, increasing the seed coat permeability for oxygen, water, and other nutrition species, consequently improving the seed growth characteristics (Zhang and Kirkham, 1994). Besides ROS and RNS, intracellular Ca^2+^ is also recognized as an important signaling molecule for many physiological processes in plants, such as hormone secretion, seed germination, cell division, cell expansion, pollen tube growth, and fertilization (Demidchik et al., 2018). Moreover, as an essential nutrient for plants, calcium deficiency or excessive calcium concentration can both lead to disorders in plants and humans (White and Broadley, 2003). However, there is still little available information about the effects of plasma on intracellular Ca^2+^ during seed germination and seedling development.

Considering the above factors, the intracellular redox state, Ca^2+^ level, and physicochemical modification of seed surface were all involved in this study to comprehensively unravel the physio-biochemical mechanism of plasma-regulated seed germination and early seedling growth using a plant model organism *Arabidopsis thaliana*.

## Materials and Methods

### Plant Material and Growth Conditions

The wild-type *Arabidopsis thaliana* seeds (Columbia-0, col) with different air-dry storage times at room temperature after harvest were used for plasma treatment. The seeds after 10-month storage were considered as the dormant seeds, while the seeds after 1-month storage were considered as the non-dormant seeds, which were only used for germination test. The seeds are collected from various plants when they are mature at the same time. Then the seeds were randomly divided into three parts for repeated testing (30–50 samples per group). *Arabidopsis* seeds were treated with sterilized water (75% ethanol: 30% hydrogen peroxide = 4:1) for 30 s and dried off naturally. Then the seeds were grown in petri dishes containing 1/2 Murashige and Skoog mineral salts (MS) medium (Sigma) including 1% (w/v) agar, 1% (w/v) sucrose (pH 6.2–6.4) and required vernalization in the dark at 4°C during 48 h. The incubating conditions were at 23°C under fluorescent light 140 ± 20 µmolm^−2^s^−1^ with 16/8 h light–dark cycle (Chen et al., 2015; Li et al., 2015; Qi et al., 2015).

### Non-Thermal Plasma System and Treatment Conditions

**Figure 1:**
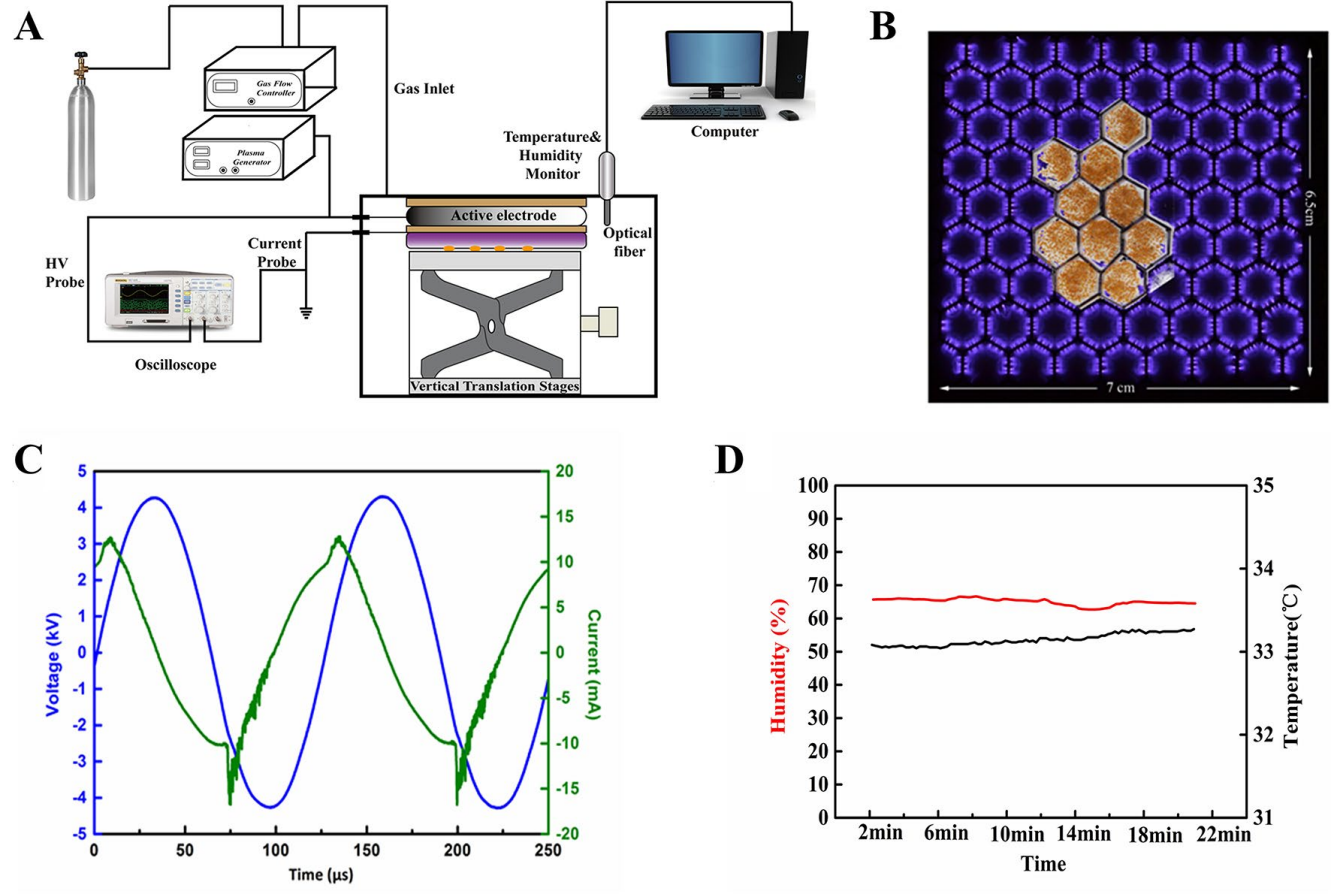
**(A)** Schematic diagram of the experimental setup used in this study. **(B)** Photo of air surface discharge treatment of *Arabidopsis* seeds. **(C)** Waveforms of the applied voltage and the discharge current. **(D)** Profiles of temperature and humidity in the atmospheric pressure discharge reactor.

**Figure 1A** shows the schematic diagram of the experimental setup. A dielectric barrier surface discharge device was used to generate the atmospheric non-thermal plasma, consisting of a copper plane electrode, a fiberglass dielectric layer, and a copper hexagonal mesh electrode. The mesh electrode was grounded and the plane electrode was connected to a 7.95 kHz high-voltage alternating-current power supply. The surface plasmas were generated in the hexagon mesh elements with a good mesh-to-mesh homogeneity (**Figure 1B**). The waveforms of discharge voltage and current were recorded by an oscilloscope. The plasma device was placed in an acrylic chamber to offer a stable gas environment for discharging. The temperature and humidity of the chamber during experiment were detected by a temperature-humidity sensor. More details of the plasma device can be found in reference (Gao et al., 2017). Air in the chamber was used as the working gas. As shown in **Figure 1C**, the applied discharge voltage had a peak-to-peak value of 8.47 kV, corresponding to a discharge power of 2.5 W. The humidity was kept at about 65% RH, and the temperature was slightly increased from 33.08 to 33.27°C during 20-min plasma discharge (**Figure 1D**).

The seeds were fixed on a quartz plate (5 cm × 5 cm) in a shape of the hexagonal mesh electrode with double-sided tape (**Figure 1B**), and closely placed under the mesh electrode. *Arabidopsis thaliana* seeds were treated by surface air plasma for 0, 0.5, 1, 3, 5, and 10 min, respectively. The seeds without plasma treatment were served as a control check (ck).

### Optical Emission Spectroscopy

Optical emission spectroscopy (OES) in the 200–800 nm range was employed to analyze the major excited reactive species in non-thermal plasma by AvaSpec-2048-8 Fiber Optic Spectrometer (Avantes, USA) (Ji et al., 2015; Yu et al., 2015).

### Measurement of Seed Germination and Seedling Growth

A seed was considered as germinated when the radicle elongated to 2 mm. The germination was counted every 12 h for 3 days. On the fifth day of germination, the primary roots of control and non-thermal plasma-treated seeds vertically cultured in petri dish were photographed with a digital camera (Nikon D5100). The length of primary roots was measured using the Image J software with the Segmentation Line tool after setting scales. The whole seedlings of 7-day-old were collected for fresh weight measurement (Zhang et al., 2016).

### Measurement of Reactive Nitrogen Species and Reactive Oxygen Species in *Arabidopsis thaliana*

The contents of RNS (NO, NO_2_^−^, NO_3_^−^, ONOO^−^) and ROS (O_2_^•−^, H_2_O_2_, .OH) in *Arabidopsis* seeds immediately after plasma treatment (day-0) and 7-day-old *Arabidopsis* seedlings germinated from the plasma-treated seeds (day-7) were detected. To test the effect of plasma treatment on NO, NO_2_^−^, NO_3_^−^, ONOO^−^, O_2_^•−^, H_2_O_2_, and .OH content in *Arabidopsis* seeds and seedlings, three different tissue extracts were carried out under conditions of different test purposes.

NO_2_^−^ Contents Were Measured Using the Method of Griess Assay, Which Involves a Diazo-Coupling Procedure (Griess, 1879). Samples (0.5 G) Were Ground in 5 Ml of 50 Mm Ice-Cold Phosphate Buffer Solution (Ph 7.8); These Samples Were Centrifuged At 12,000 Rpm/Min for 10 Min At 4°C. The Supernatant Was Used for Determining the Content of NO, NO_2_^−^, and NO_3_^−^. NO_2_^−^ reacts with sulfartilic acid under acidic conditions to form a diazoniurn cation, which subsequently couples to the aromatic amine 1-naphthylamine to produce a red-violet colored (540 nm), water-soluble azo dye. in this process, the total NO content can be represented indirectly by adding nitrate reductase (Beyotime, S0024), and NO_3_^−^ can be directly measured by UV absorbance detection at 210–220 nm (Wang et al., 2017). a standard curve was plotted with known concentrations of NO, NO_2_¯, and NO_3_¯ t o measure their concentration in the experimental samples.

ONOO ¯ was determined according to the method of Huang et al. (2007). Folic acid was prepared by dissolving appropriate amount of folic acid in 0.001 mol/L NaOH and kept frozen, and the working barbital buffer solution was prepared by dissolving 4.125 g barbital sodium in 500 ml distilled water and add 0.7 ml 1.0 mol/L HCl. 0.2 g samples were collected and ground in 2 ml alcohol, centrifuged at 12,000 rpm/min for 10 min at 4°C; the supernatant was used for determining the content of ONOŌ. An aqueous solution of 1 ml folic acid was rapidly mixed with 3 ml working barbital buffer solution and then 300 µl seeds or seedling extracts were immediately added for 5 min at room temperature. High fluorescence product was generated by the introduction of peroxynitrite into the solution of folic acid, resulting in dramatic increase in spectra characteristics with excitation maximum at 380 nm and fluorescence emission maximum at 460 nm.

The H_2_O_2_ content was measured by the method of Wang et al. (2017a). A total of 0.2 g samples (seeds or 0.2 g of fresh weight (FW) from 7-d-old seedlings) were extracted in 5 ml of pre-cooled acetone at 4°C ice bath, centrifuged at 10,000 rpm/min for 15 min at 4°C. The supernatant was removed and reacted with 0.1 ml mixture solution of titanium tetrachloride (TiCl_4_) in 10% hydrochloric acid (HCl) (v/v). Reaction for 5 min at room temperature (25°C), the mixture was centrifuged at 8,000 rpm/min for 10 min at 4°C. The precipitate was washed 3–5 times with cold acetone and dissolved in 3 ml 1 M H_2_SO_4_. Absorption at 410 nm was recorded (UV mini-1240, SHIMADZU, Japan), and the H_2_O_2_ concentration was calculated from a standard curve prepared in the same manner.

The most frequently used approach to detect O_2_^•−^ is the nitroblue tetrazolium (NBT) colorimetric (Jambunathan, 2010). Nitroblue tetrazolium chloride (0.1% [w/v] NBT was prepared in 10 mM sodium azide, 50 mM potassium phosphate. 0.5 g samples were ground in 5 ml of the above solution, centrifuged at 12,000 rpm/min for 10 min at 4°C; the supernatant absorbance of the samples was read at 580 nm.

The spectral characteristics of hydroxyl groups were detected using attenuated total reflectance fourier transform infrared measurements (ATR-FTIR) in the spectral range of 4,000–400 cm^−1^ (NICOLET IS10, Thermo Fisher Scientific, USA) (Sharma and Uttam, 2019). The absorption band 3,700–3,100 cm^−1^ ascribed with -OH stretching confirmed the presence of chemical compounds with hydroxyl groups (Mishra and Mohanty, 2019). 0.2 g samples were collected and ground in 2 ml alcohol, centrifuged at 12,000 rpm/min for 10 min at 4°C, and the supernatant was used for determining the intensity of hydroxyl groups. **Supplemental Figure 1** indicates that the intensity of the absorption band 3700–3100 cm^−1^ ascribed with hydroxyl group can characterize the level of hydroxyl radicals in seeds, which was detected by terephthalic acid (TA) method (Barreto et al., 1994).

### Measurement of Lipid Peroxidation and Antioxidants in *Arabidopsis thaliana*

The contents of malondialdehyde (MDA) and antioxidants (proline, CAT, SOD and POD) in *Arabidopsis* seeds immediately after plasma treatment (day-0) and 7-day-old *Arabidopsis* seedlings germinated from the plasma-treated seeds (day-7) were detected.

MDA is the end product of lipid peroxidation and reflects the degree of damage due to adverse conditions. The levels of lipid peroxidation in *Arabidopsis* were determined by the trichloroacetic acid (TCA) reaction (Shalata and Neumann, 2001). Samples of seeds or seedlings (0.2 g) were collected and ground sufficiently with 5 ml 10% TCA and a little of SiO_2_. After centrifugation at 5,000 rpm/min for 10 min, the supernatant (2 ml) was combined with 0.6% (w/v) thiobarbituric acid (2 ml) and incubated in boiling water for 15 min. The reactions were stopped by placing the tubes in an ice bath. The mixture was centrifuged at 5,000 rpm/min for 15 min and the supernatants were read at 532 and 450 nm.

The proline content was measured using colorimetric methods according to (Bates et al., 1973). The irradiated samples 0.2 g seeds and 0.2 g FW of 7-day-old whole seedlings germinated from irradiated seeds were homogenized in liquid nitrogen. Tissues were suspended in 5 ml of 3% sulfosalicylic acid and incubated in boiling water for 10 min. After centrifugation, 2 ml supernatant was mixed with 4 ml acid ninhydrin, 2 ml acetic acid and 2 ml distilled water to a final volume of 10 ml. All the mixtures were incubated in boiling water for 1 h. The reaction mixtures were then extracted with 4 ml toluene, and the upper phases were collected. The absorbance of samples was read at 520 nm, and a standard curve was plotted with known concentrations of proline to measure the proline concentration in experimental samples.

Both seeds (day-0) and seedlings (day-7) were homogenized in a mortar with 2 ml of 50 mM ice-cold phosphate buffer (pH 7.8) containing 1 mM EDTA. The homogenate was collected and centrifuged at 12,000 rpm/min for 15 min at 4°C. The supernatant was used for determining the activities of CAT, SOD and POD based on the Refs (Zhang and Kirkham, 1994; Jiang et al., 2014; Qi et al., 2015), respectively. All procedures were performed at 4°C.

The activity of CAT was determined using the method that involves measuring the initial rate of disappearance of H_2_O_2_, as described (Qi et al., 2015). The enzyme extract (0.1 ml) which was added to the reaction solution (3 ml) consisted of 50 mM phosphate buffer (pH7.0), 20 mM H_2_O_2_. A decrease in H_2_O_2_ was monitored at 240 nm for at least 3 min.

The SOD activity was determined using the nitro-blue tetrazolium (NBT) method (Zhang and Kirkham, 1994). 3 ml reaction mixture contained 50 mM phosphate buffer (pH7.8), 0.1 mM EDTA, 130 mM methionine, 0.75 mM NBT, 0.02 mM riboflavin, and 0.1 ml enzyme extract. Riboflavin was added as the last component, and the reaction mixtures were illuminated for 15 min at a light intensity of 5,000 lx. Non-illuminated and illuminated reactions without the supernatant served as calibration standards. One unit of SOD activity was defined as the amount of enzyme required to cause 50% inhibition of the reduction of NBT monitored at 560 nm.

The guaiacol method was used to measure the POD activity (Jiang et al., 2014). The enzyme extract (0.02 ml) was added to the reaction mixture containing 0.02 ml guaiacol solution and 0.01 ml H_2_O_2_ solution in 3 ml of phosphate buffer solution (pH 7.0). The addition of the enzyme extract started the reaction, and the increase in absorbance was recorded at 470 nm for 5 min.

### Assessment of Intracellular Ca^2+^

The Ca^2+^ content was tested using the probe fluorescein Fluo-2-acetoxymethyl ester (Fura-2 AM) (S1052, Beyotime). The 4-day-old seedlings were stained with 0.5 µM Fura-2 AM for 45 min at room temperature (25°C), and washed with fresh liquid media three times before observation. All the experiments were captured for six different fields. The images were taken by fluorescence microscope (Leica DM4000B, Germany).

### Scanning Electron Microscopy-Energy Dispersive X-ray Spectroscopy

The seeds were dried completely under the ethanol dehydration (20%, 40%, 60%, 80%, 100%; 15 min each time), and then the surface was sputter-coated with gold and observed by scanning electron microscopy (SEM) (JSM-6700F/INCA-ENERGY, JEOL, Japan) (Chen et al., 2012). The elements on seed surface were analyzed by energy dispersive X-ray (EDX) spectroscopy with a X-MaxN (51-XMX1146) (Gómez-Ramírez et al., 2017).

### Statistical Analysis

Experiments were performed using a completely randomized designs with three independent replicate experiments (n = 3). Differences were analyzed with One-way analysis of variance (ANOVA) and least significant difference (LSD) test. Analyses were performed using SPSS statistical software package (SPSS Inc., version 17.0, USA). Differences at *p* < 0.05 were considered significant.

## Results and Discussions

### The Major Excited Species in Air Plasma by Optical Emission Spectroscopy

**Figure 2:**
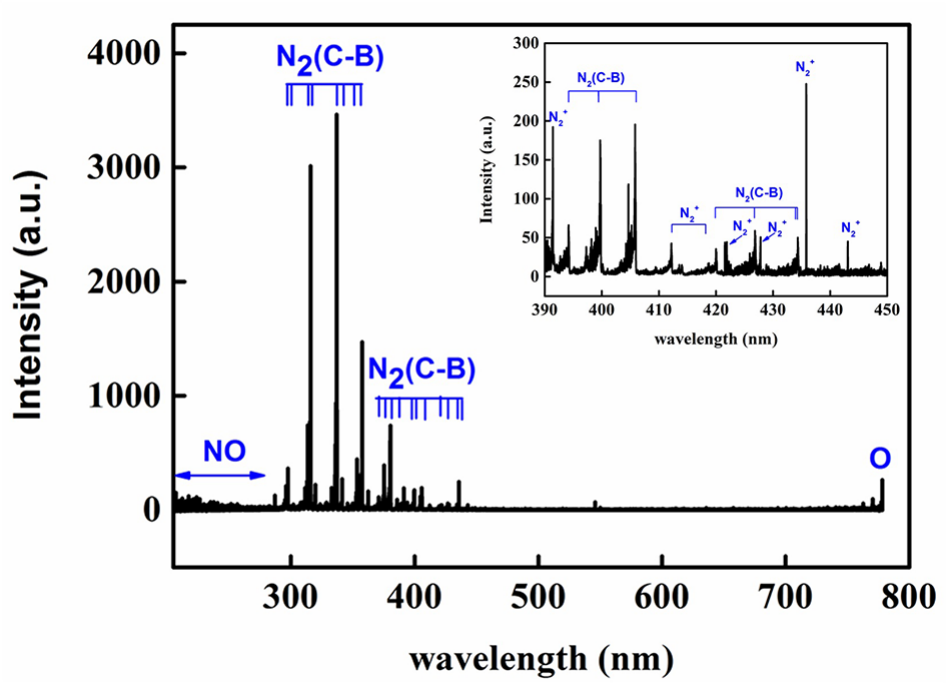
End-on optical emission spectrum of the air plasma in the range of 200−800 Nm.

OES was used to investigate the excited active species generated in the surface air plasma ranging from 200 to 800 nm. As observed in **Figure 2**, the spectra was dominated by N_2_ second positive system (C^3^∏_u_→B^3^∏_g_) emissions at 297.6, 313.6, 315.9, 333.8, 337.1, 353.7, 357.7, 371.0, 375.5, 380.5, 385.8, 394.3, 399.8, 405.9, 420.1, 427.0, and 434.3 nm as well as by N_2_^+^ first negative system (B^2^∑_u_→X^2^∑_g_) emissions at 391.0 and 400–450 nm as a result of the direct or step-wise electron impact excitations and the pooling reaction of the nitrogen metastable state (Yu et al., 2015). The NOγ-system (A^2^∑^+^→X^2^∏) emission bands from 200 to 280 nm and atomic oxygen emission at 777.2 nm were also observed. The OES results indicated that abundant excited nitrogen molecules and ion, atomic oxygen and NO existed in the air plasma, which can react with each other to form various kinds of RNS and ROS (hydroxyl [OH], hydrogen peroxide [H_2_O_2_], nitrite [NO_2_^−^], nitrate [NO_3_^−^], ozone [O_3_], etc.) due to their high activity (Liang et al., 2012). These reactive species could not only react with the seed coat of *Arabidopsis*, but also regulate its growth and development as secondary messengers.

### Effect of Plasma on Seed Germination and Seedling Growth

**Figure 3:**
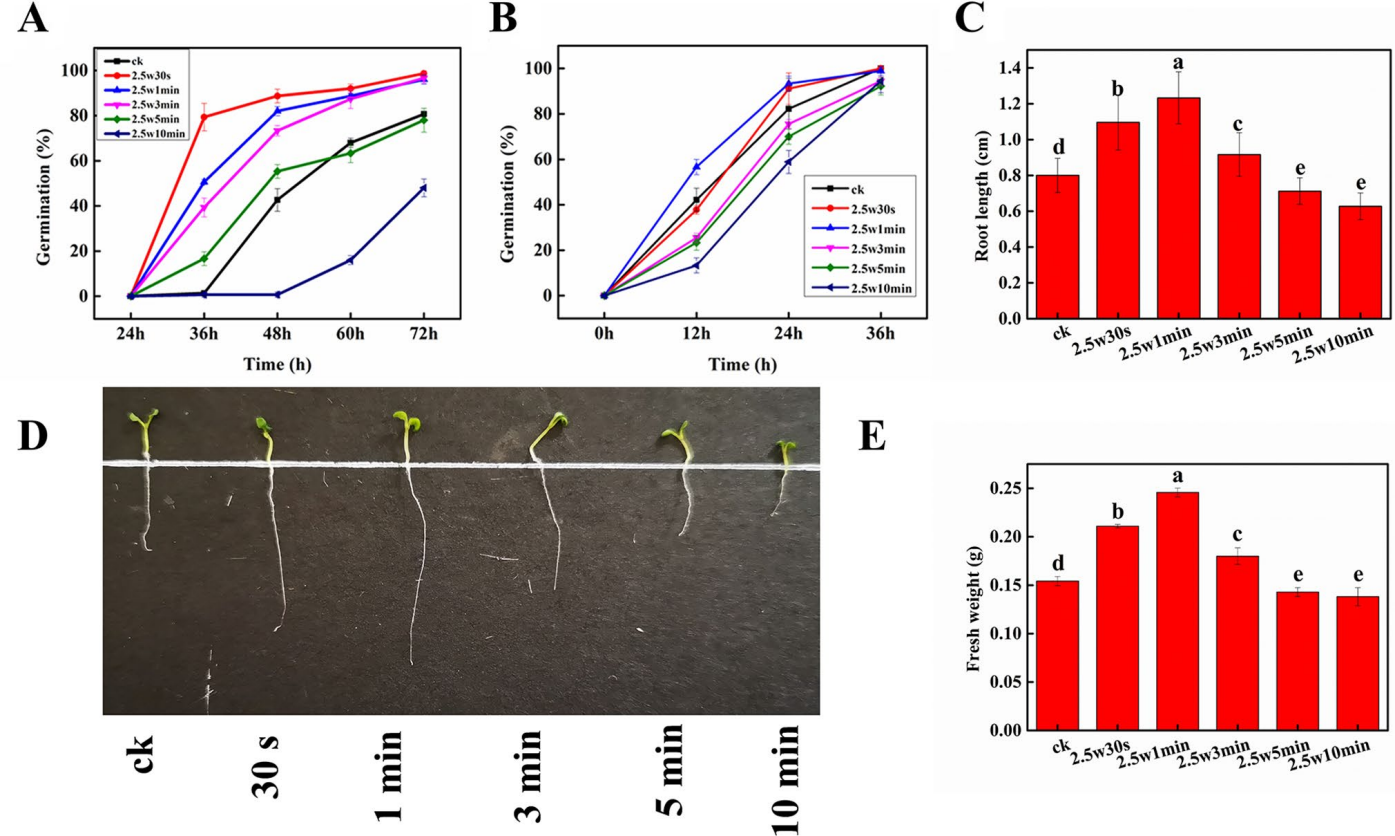
Effect of plasma on germination of dormant **(A)** and non-dormant **(B)** seeds, radical length **(C**, **D)** and fresh weight **(E)** of *Arabidopsis* seeds. Different letters (a–e) indicate significant differences among treatments according to LSD test (*p* 0.05).

**Figure 3A** shows the germination rate of dormant *Arabidopsis* seeds exposed to non-thermal plasma at different times during 3-day cultivation. On day 3, about 20% seeds still can’t germinate in the control group, while the seeds after short-time plasma treatment (0.5, 1, and 3 min) almost completely germinated (98.7%, 96.0%, 96.7%), indicating that plasma could break the seed dormancy in *Arabidopsis*. Similar results were also reported by Šerá et al. (2009), who found that plasma can break seed dormancy and accelerate seed germination in Lamb’s Quarters seeds. It is generally accepted that the elevated ROS and RNS production in the embryos is necessary for seed transition from the dormant to the non-dormant state, and to accomplish the germination process. Thus, the plasma-generated ROS and RNS may mainly contribute to the seed dormancy alleviation in *Arabidopsis* seeds. For long-time plasma treatment, 5-min plasma exposure initially promoted the seed germination, then slightly inhibited after 48 h. 10-min plasma exposure significantly inhibited the seed germination, suggesting that the plasma either reduces the seed viability or deepens their dormancy. And previous studies have suggested that a prolonged plasma treatment or a treatment with more reactive and energetic plasma active species would have negative effects on germination (Bafoil et al., 2019). For the non-dormant *Arabidopsis* seeds (**Figure 3B**), the final germination of untreated seeds rose to almost 100% after 36-h cultivation, while 0.5 and 1-min plasma exposure resulted in the same final germination rate and higher germination speed compared with that of control, indicating that besides seed dormancy release, the germination-promoting effects of non-thermal plasma may be also attributed to other factors such as the modification of seed coat (Volin et al., 2000), improvement of wettability and water imbibition of seeds (Jiang et al., 2014; Bormashenko et al., 2015), as well as the increase of soluble sugar and soluble protein concentrations in seeds (Henselová et al., 2012).

Root length and fresh weight were measured to estimate the effect of non-thermal plasma on *Arabidopsis* plant growth. As shown in **Figures 3C–E**, during 10-min plasma treatment, the values of root length and fresh weight all peaked at 1 min, and then decreased with longer plasma treatment time. Compared with untreated seedlings, 0.5, 1, and 3-min plasma treatment led to a distinct increase of 36.9, 53.9, and 14.5% in root length as well as 36.7, 59.2, and 16.7% in fresh weight (*p* < 0.05), respectively. While a decrease of 11.0 and 21.6% in root length as well as 7.3 and 10.4% in fresh weight (*p* < 0.05) was observed in 5 and 10-min treated seedlings, respectively. These results indicated that short-time plasma exposure (≤3 min) could enhance the seed germination and seedling growth of *Arabidopsis*, but long-time plasma treatment (≥5 min) exhibited inhibitory effects. This phenomenon is so-called dose-dependent effect induced by non-thermal plasma, which has also been observed in various plants (Guo et al., 2017).

### Effect of Plasma on Reactive Oxygen Species and Reactive Nitrogen Species in *Arabidopsis*

**Figure 4:**
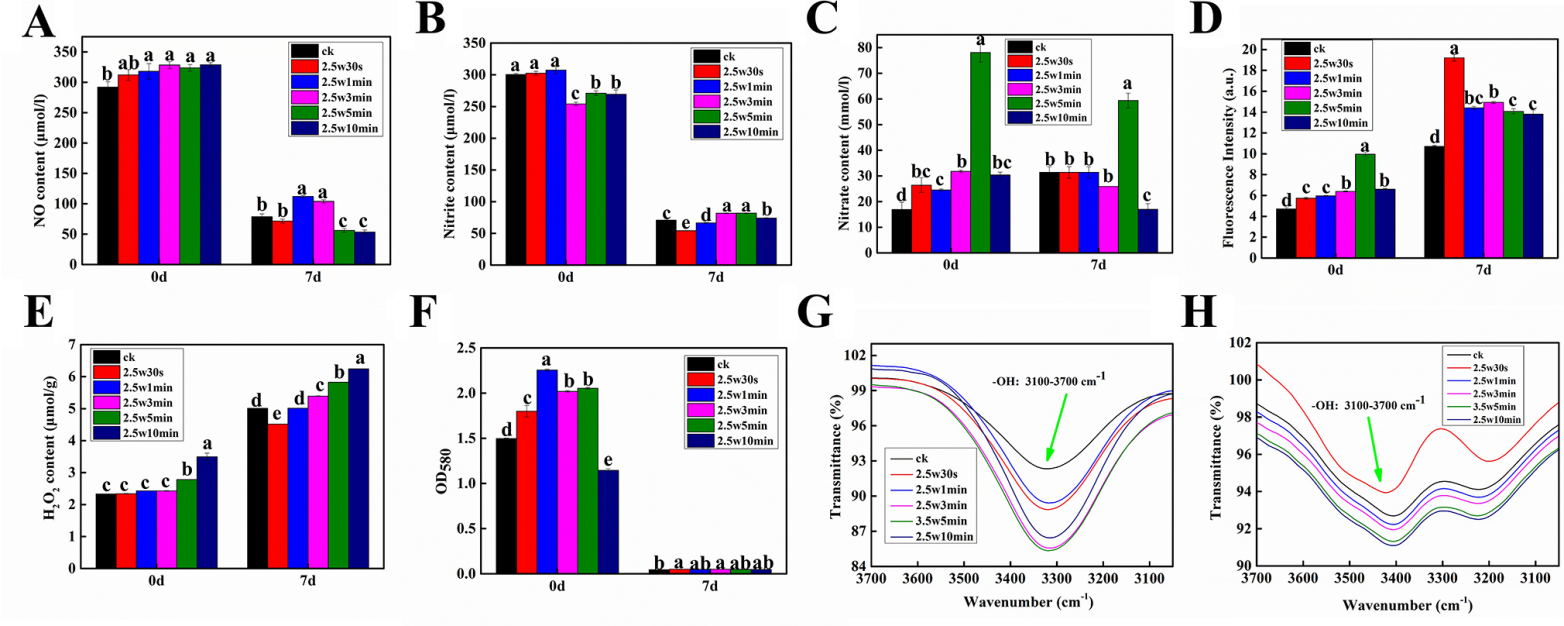
Effect of plasma on the level of NO **(A)**, nitrite **(B)**, nitrate **(C)**, peroxynitrite anion **(D)**, H_2_O_2_ **(E)**, superoxide anion **(F)** as well as the intensity of infrared absorption of hydroxyl group **(G**, **H)** in *Arabidopsis* seeds immediately after plasma treatment (day-0) and 7-day-old *Arabidopsis* seedlings germinated from the plasma-treated seeds (day-7).

Given that ROS and RNS as the signal molecules play important roles in seed dormancy alleviation, seed germination and seedling growth (Singh et al., 2016), seven RNS and ROS (NO, NO_2_ ¯, NO_3_ ¯, ONOO ¯, O_2_^•−^, H_2_O_2_, and OH) were measured in *Arabidopsis* seeds immediately after plasma treatment (day-0) and 7-day-old *Arabidopsis* seedlings germinated from the plasma-treated seeds (day-7) to investigate the effect of non-thermal plasma on the level of ROS and RNS during seed germination and seedling growth in *Arabidopsis*. As shown in **Figure 4**, except for NO_2_ ¯, the other ROS and RNS contents in *Arabidopsis* seeds were all increased compared with untreated samples, indicating that the reactive species produced by plasma could permeate into the interior of seeds through the seed coat. The levels of NO and H_2_O_2_ in *Arabidopsis* seeds were increased with plasma treatment time (**Figures 4A, E**), while the level of O_2_^•−^ and the intensity of infrared absorption of hydroxyl group were all increased firstly and then decreased with prolonged plasma treatment time (**Figures 4F, G**), indicating that the short-lived ROS (O_2_^•−^ and OH) may convert into the long-lived ROS (H_2_O_2_) for long-time plasma treatment, consequently leading to a markedly higher H_2_O_2_ content in 5 and 10-min treated seeds. For example, O_2_^•−^ can convert into H_2_O_2_ and O_2_ by SOD, and the excessive OH can form H_2_O_2_*via* the reaction (OH + OH → H_2_O_2_) (Zhang et al., 2018). For NO_2_^−^ contents, there was no significant difference among 0, 0.5, and 1-min treated seeds, while 3, 5, and 10-min plasma treatment significantly decreased NO_2_^−^ contents(**Figure 4B**). The NO_3_ ¯ contents ONOO ^−^ contents had similar change trend, which was increased in the first 5 min and significantly reduced at 10 min (**Figures 4C, D**). Combined with the results of germination, it is concluded that O_2_^•−^, OH, and NO induced by plasma can break seed dormancy and accordingly improve the seed germination in short-time plasma treated seeds, while high H_2_O_2_ concentration can cause oxidative damages in long-time plasma treated seeds, consequently inhibiting the seed germination. Similar results were also reported by Whitaker et al. (2010), who found that reagents that generate OH and O_2_^•−^ can break seed dormancy in *Bidens pilosa* L. seeds, while H_2_O_2_ tended to inhibit rather than promote total germination, indicating that it is OH and O_2_^•−^ rather than H_2_O_2_ implicated in the release of seed dormancy in this species. For *Arabidopsis* seedlings, the level of H_2_O_2_ and the intensity of infrared absorption of hydroxyl group had similar change pattern, which were all significantly increased with the plasma treatment time except for 0.5-min treated seedlings (**Figures 4E, H**). These results suggested that moderate H_2_O_2_ and OH have positive effects on seedling growth of *Arabidopsis*, while excessive H_2_O_2_ and OH could inhibit the seedling growth. The O_2_^•−^ contents in *Arabidopsis* seedlings were very low compared with that of seeds (**Figure 4F**), indicating that O_2_^•−^ almost has no effects on the seedling growth. Compared with untreated seedlings, the NO concentrations in 1 and 3-min treated seedlings were significantly increased, while 5 and 10-min plasma treatment significantly decreased the NO concentration, indicating that NO also plays an important role in improving the seedling growth after short-time plasma treatment.

### Effect of Plasma on Lipid Peroxidation and Antioxidants in *Arabidopsis*

**Figure 5:**
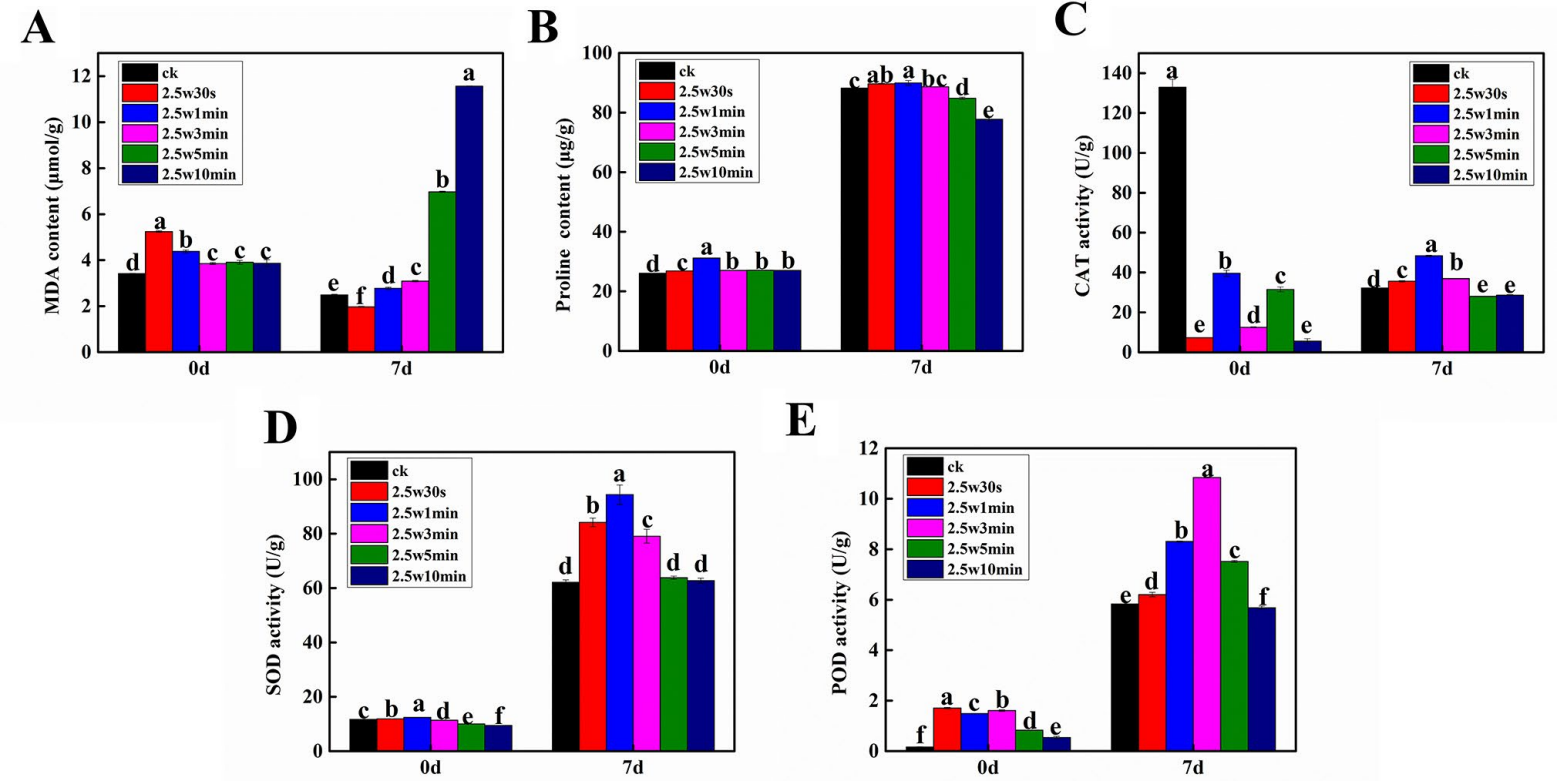
Effect of plasma on the level of MDA **(A)** and proline **(B)** as well as the activity of CAT **(C)**, SOD **(D)** and POD **(E)** in *Arabidopsis* seeds immediately after plasma treatment (day-0) and 7-day-old *Arabidopsis* seedlings germinated from the plasma-treated seeds (day-7).

Due to the fact that the intracellular redox state is determined by ROS and antioxidants and the cell would suffer oxidative stress when the antioxidants cannot scavenge excessive ROS (Kumar and Prasad, 2018), four typical antioxidants (proline, CAT, SOD, and POD) and a commonly used oxidative stress indicator (MDA) were measured in *Arabidopsis* seeds immediately after plasma treatment (day-0) and 7-day-old *Arabidopsis* seedlings germinated from the plasma-treated seeds (day-7) to evaluate the effects of non-thermal plasma on the cellular redox homeostasis of *Arabidopsis*. SOD, POD, and CAT together form the first and most important line of antioxidant enzyme defense systems against ROS. SOD catalyzes the dismutation of O_2_^•−^ to H_2_O_2_ and O_2_, then both CAT and POD decompose H_2_O_2_ into H_2_O and O_2_ (Sun et al., 2014). Proline can scavenge OH as a non-enzymatic antioxidant (Soares et al., 2016). As shown in **Figure 5A**, the MDA level was significantly increased in plasma-treated seeds compared with the untreated seeds. The proline level and SOD and POD activity in *Arabidopsis* seeds were all increased firstly and then decreased with the plasma treatment time (**Figures 5B, D, E**). The CAT activity in plasma-treated seeds was significantly decreased compared with the untreated seeds (**Figure 5C**). However, we didn’t obtain a clear change law of these parameters following the change of plasma treatment time, which may due to the fact that these parameters didn’t have enough time to respond adequately to the ROS and RNS in seeds when they were immediately measured after plasma treatment.

For *Arabidopsis* seedlings, the concentration of MDA was increased with the plasma treatment time. Except for 0.5-min treated seedlings, the MDA level was higher than in untreated samples after plasma exposure (**Figure 5A**). Especially, 5 and 10-min treatments caused a dramatic increase of 180.0% and 364.3% in MDA content. These results revealed that short-time plasma treatment (≤3 min) only slightly elevated the oxidative level in *Arabidopsis* seedlings, while the long-time treated seedlings (≥5 min) were subjected to severe oxidative stress. In **Figures 5B–E** all significantly enhanced relative to untreated samples, which is due to the elevated oxidative stress caused by plasma. Similar results have also been reported by other groups in various plants. Puač et al. (2014) showed that plasma induced higher SOD and CAT activity in meristematic cells of *Daucus carota* both immediately and 2 weeks after plasma treatment. Hosseini et al. (2018) found that nitrogen plasma increased POD and CAT activity in artichoke seeds. Zahoranová et al. demonstrated that SOD, CAT, and guaiacol peroxidase (POX) activity of maize seeds were enhanced by plasma (Zahoranová et al., 2016). Besides 5-min treated seedlings having a higher POD activity compared with control, the long-time plasma treatment (≥5 min) either caused a significant decrease in the proline content and activity of CAT and POD or had no effects on the SOD activity. These results reveal that the alteration of redox state may play important roles in plasma-regulated seedling growth of *Arabidopsis*. It is hypothesized that the antioxidant systems with enhanced activities can effectively scavenge the slightly increased ROS in *Arabidopsis* seeds after short-time plasma exposure (≤3 min), consequently leading to growth promotion. Conversely, the long-time plasma treated seedlings (≥5 min) didn’t have enough antioxidants to combat with the severe oxidative stress, accordingly displaying inhibited seedling growth.

### Effect of Plasma on Intracellular Ca^2+^

**Figure 6:**
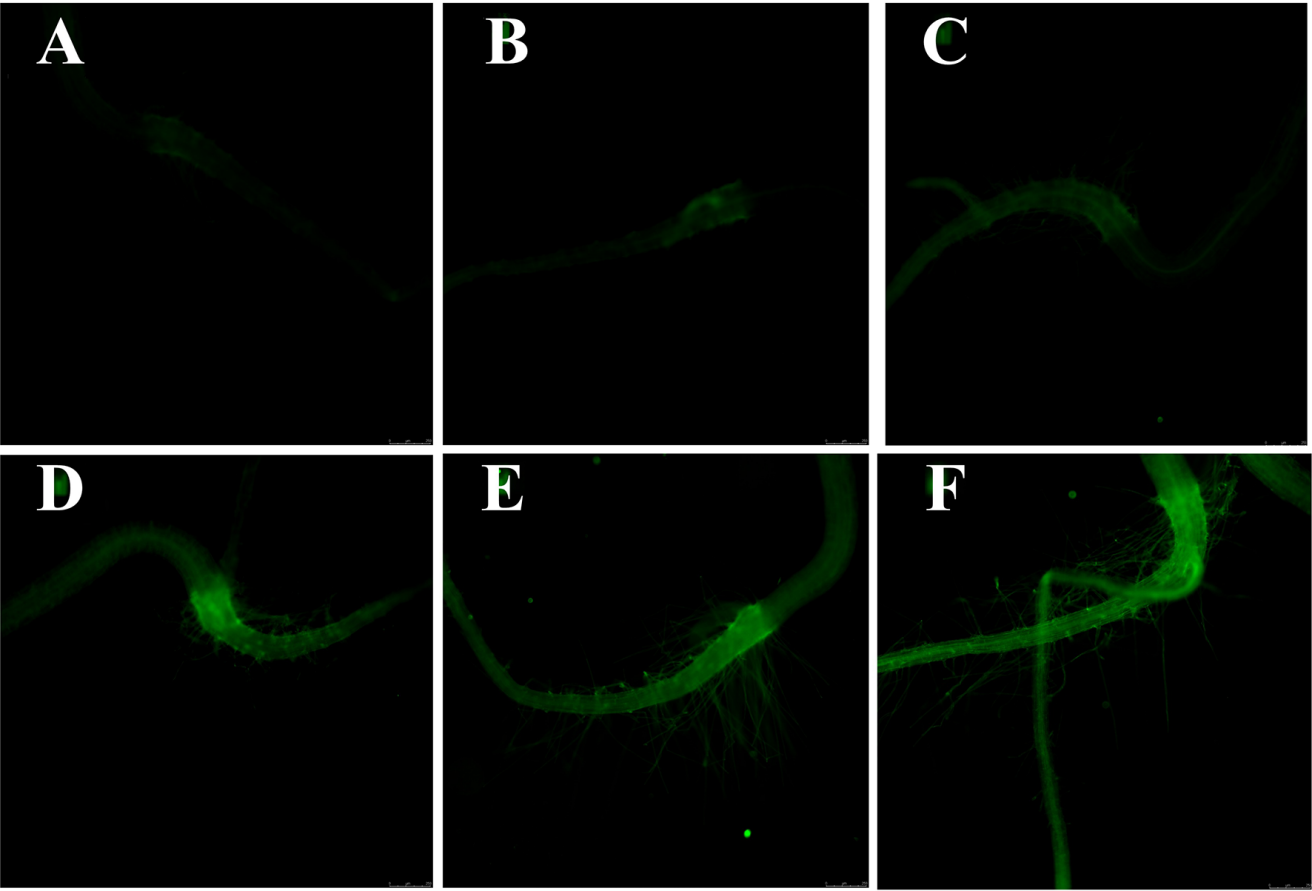
The cytosolic Ca^2+^ fluorescence microscope images (10×) of **(A)** 0, **(B)** 0.5, **(C)** 1, **(D)** 3, **(E)** 5, and **(F)** 10-min plasma treated *Arabidopsis* seedlings. The magnification for all images was 5×.

Given that Ca^2+^ is not only a necessary nutrient for plants, but also an important signal molecule for many physiological processes, the intracellular Ca^2+^ level of *Arabidopsis* seedlings was measured with calcium-specific fluorescent dye (Fluo-2 AM) *via* fluorescence microscope. Several studies have shown that excessive tissue calcium concentration will result in cellular toxicity, in overly rigid cell walls and in developmental abnormalities (Conn et al., 2011; Cybulska et al., 2011). As presented in **Figure 6**, the green fluorescence intensity was significantly increased with the plasma treatment time, indicating that plasma led to the Ca^2+^ accumulation in *Arabidopsis* seedlings. Similar to ROS, Ca^2+^ also has dose-dependent effects on plants. Low level of Ca^2+^ promotes plant growth, while excessive Ca^2+^ causes cytotoxicity to plants *via* affecting the ion homeostasis and osmotic pressure (Volk et al., 1997). High calcium concentration in plant tissue can result in cellular toxicity or developmental abnormalities (Hocking et al., 2016). Considering the plant growth results, it is concluded that intracellular Ca^2+^ may also participate in the plasma-regulated seedling growth of *Arabidopsis*.

### Effect of Plasma on the Physicochemical Modification of Seed Surface

**Figure 7:**
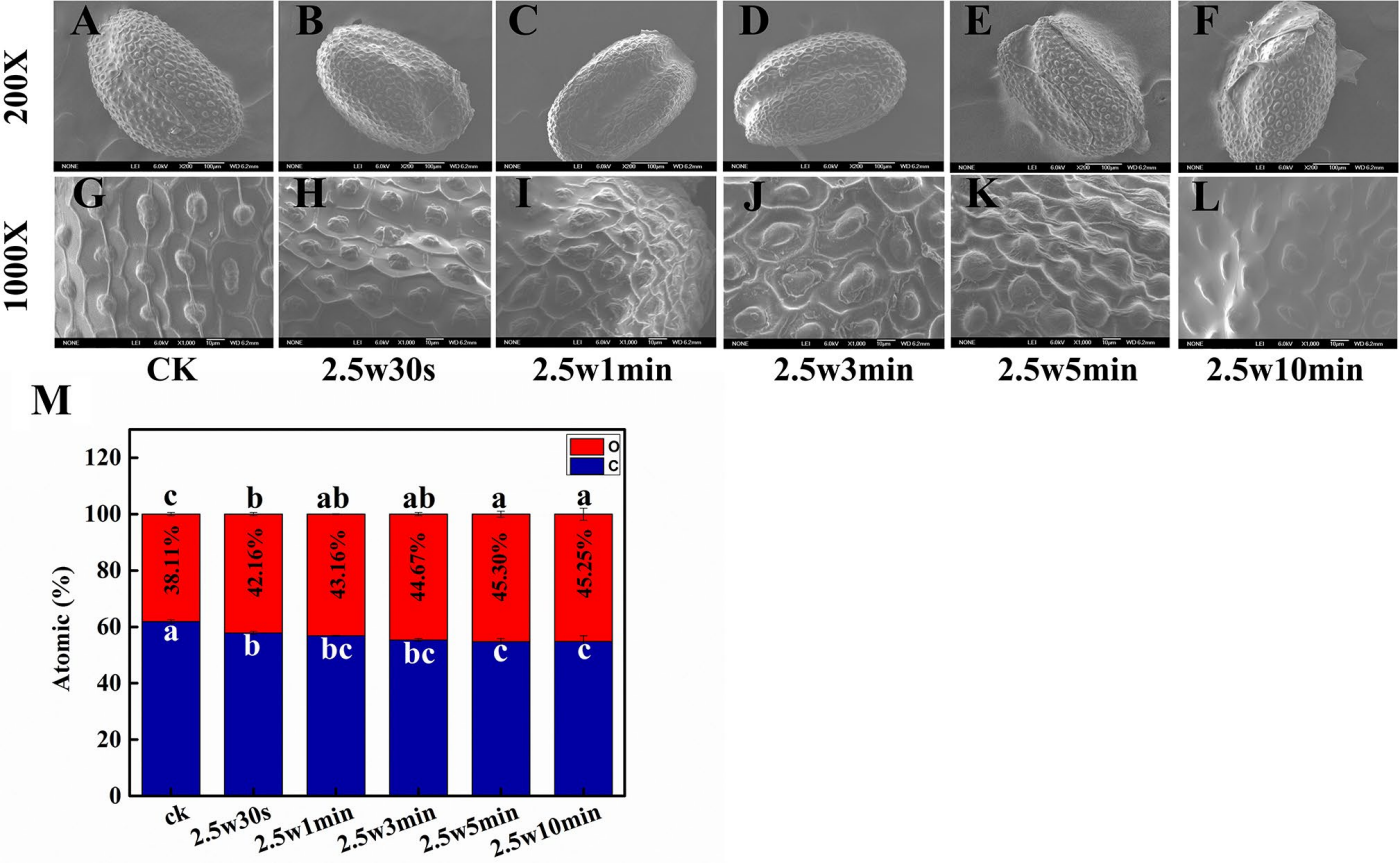
SEM images of **(A**, **G)** 0, **(B**, **H)** 0.5, **(C**, **I)** 1, **(D**, **J)** 3, **(E**, **K)** 5 and **(F**, **L)** 10-min plasma treated *Arabidopsis* seed surface. The magnification for all images was 200× (top) and 1000× (bottom). **(M)** Effect of plasma on atomic ratio of C and O in *Arabidopsis* seed surface.

The structural and chemical changes of seed coat after plasma treatment were observed by scanning electron microscopy-energy dispersive X-ray spectroscopy (SEM-EDX). SEM images show that the seed husk of *Arabidopsis* undergoes physical distortion to varying degrees with the increasing plasma treatment time (**Figures 7A–L**). There were no visible morphological alterations after 0.5-min plasma treatment. However, the epidermis was obviously deformed and shrinked in 1 and 3-min treated seeds. After 5-min plasma treatment, the epidermis was detached from the seed. For 10-min plasma treated seeds, the seed surface was even smoothed without the protection of epidermis. Plasma-induced etching effects on seed surface are closely related to the seed type and surface morphology (Butscher et al., 2016). *Arabidopsis* seed epidermis with a highly compact surface texture is more fragile and easier to be detached from the seed surface after plasma treatment, which is conducive to absorbing water and nutrients in the surrounding environment for seeds, consequently enhancing the seed germination (Stolárik et al., 2015; Wang et al., 2017b). Similar results were reported by Bormashenko et al. and Khamsen et. al., who also demonstrated that the seed surface modification caused by plasma is beneficial for seed germination (Bormashenko et al., 2015; Khamsen et al., 2016).

The etching effects of plasma on seed surface were most probably attributed to the oxidation of RONS in plasma. To verify this hypothesis, the chemical composition of the seed surface after plasma treatment has also been analyzed by EDX. C and O are the main elements of the seed surface of *Arabidopsis*. As shown in **Figure 7M**, relative to untreated samples with an O ratio of 38.11%, the O ratio of seed surface was significantly increased from 42.16 to 45.25% with plasma treatment time from 0.5 to 10 min, indicating that plasma could indeed increase the oxidation degree of *Arabidopsis* seed surface. The SEM-EDX results indicated that the moderate surface deformation of *Arabidopsis* seed induced by short-time plasma treatment (≤3 min) *via* oxidative etching is a predominant mechanism for improved seed germination and seedling growth.

## Conclusion

In conclusion, plasma exhibited a dose-effect on seed germination and early seedling growth of *Arabidopsis*, ranging from stimulation (≤3 min) to inhibition (≥5 min) depended on the plasma treatment time. For *Arabidopsis* seeds, O_2_^•−^, ·OH, and NO play important roles in the improvement of seed germination, while H_2_O_2_ could inhibit the seed germination. For *Arabidopsis* seedlings, the positive effects of short-time plasma treatment are ascribed to the enhanced activities of antioxidant system, moderate intracellular ·OH, H_2_O_2_, NO, and Ca^2+^ accumulation and mild seed surface modification. Whereas, severe oxidative stress caused by high level of ·OH and H_2_O_2_, excessive intracellular Ca^2+^, and severely damaged seed surface induced by long-time plasma treatment can result in the inhibitory effects. This work extends the available knowledge of physio-biochemical mechanism underlying plasma-regulated seed germination and seeding growth in *Arabidopsis*, which would be beneficial to promote the application of non-thermal plasma as a promising pre-sowing seed approach in agriculture.

## Data Availability Statement

All datasets generated for this study are included in the article/**Supplementary Material**.

## Author Contributions

DC and RM conceived the original screening and research plans. ZJ supervised the experiments. YY, ZW, HD, and ZJ provided technical assistance to DC. DC, YY, JW, and RM designed the experiments. DC, YY, and JW performed most of the experiments. DC, YY, JW, and ZW made great contribution on data collecting and analyze. DC, RM, and ZJ contributed on interpretation of data for the work. DC conceived the project and wrote the article with contributions of all the authors. ZW, HD, and RM revised it critically for important intellectual content. ZJ supervised and complemented the writing. RM, and ZJ agrees to serve as the author responsible for contact and ensures communication.

## Funding

This work was funded by the National Natural Science Fund of China (11605159 and 11704343), Chinese Postdoctoral Science Foundation (2017M612412), the Foundation of Key Technology Research Project of Henan Province (182102110090).

## Conflict of Interest

The authors declare that the research was conducted in the absence of any commercial or financial relationships that could be construed as a potential conflict of interest.
