# Supplementary material for: Research on the Physio-Biochemical Mechanism of Non-Thermal Plasma-Regulated Seed Germination and Early Seedling Development in Arabidopsis
Source: Front Plant Sci. 2019 Nov 8;10:1322. doi: 10.3389/fpls.2019.01322 (PMC6857620; doi:10.3389/fpls.2019.01322)

**Electronic Supplementary Material for**

[**The Physio-biochemical Mechanism of Non-thermal Plasma-regulated Seed Germination and Early Seedling Development in *Arabidopsis***](http://xueshu.baidu.com/s?wd=paperuri%3A(413932b9c4d1ee52424048064d0cb040)&filter=sc_long_sign&sc_ks_para=q%3DThe GCR2 gene family is not required for ABA control of seed germination and early seedling development in Arabidopsis&sc_us=16360461045425754815&tn=SE_baiduxueshu_c1gjeupa&ie=utf-8)

**Dongjie Cui,^1^ Yue Yin,^1^ Jiaqi Wang,^1^ Hangbo Xu,^1^ Pengtao Yuan,^2^ Zhiwei Wang,^3^ Hongbin Ding,^3^ Ruonan Ma,^1,*)^ and Zhen Jiao^1,*)^**

^1^Henan Key Laboratory of Ion-beam Bioengineering, College of Agricultural, Zhengzhou University, Zhengzhou, China, 450052

^2^School of Pharmacy, East China University of Science and Technology, Shanghai, China, 200237

^3^School of Physics, Key Laboratory of Materials Modification by Laser, Ion and Electron Beams, Ministry of Education, Dalian University of Technology, Dalian, China, 116024

*Co-Corresponding author: Ruonan Ma and Jiao Zhen.

E-mail address: [mrn19890324@aliyun.com](mailto:mrn19890324@aliyun.com) and [jiaozhen@zzu.edu.cn](mailto:jiaozhen@zzu.edu.cn).

**Supplementary material captions:**

**Supplemental Figure 1.** Effect of 10-min plasma treatment on the level of hydroxyl (A) and hydroxyl radical (B) in deionized water. (A: Attenuated total reflection-Fourier-transform infrared (ATR-FTIR) spectroscopy was used to detect hydroxyl groups produced by plasma irradiation; B: Terephthalic acid (TA) as a hydroxyl radical dosimeter was used to detect hydroxyl radicals produced by plasma irradiation.)

.

**Supplemental Figure 1.**


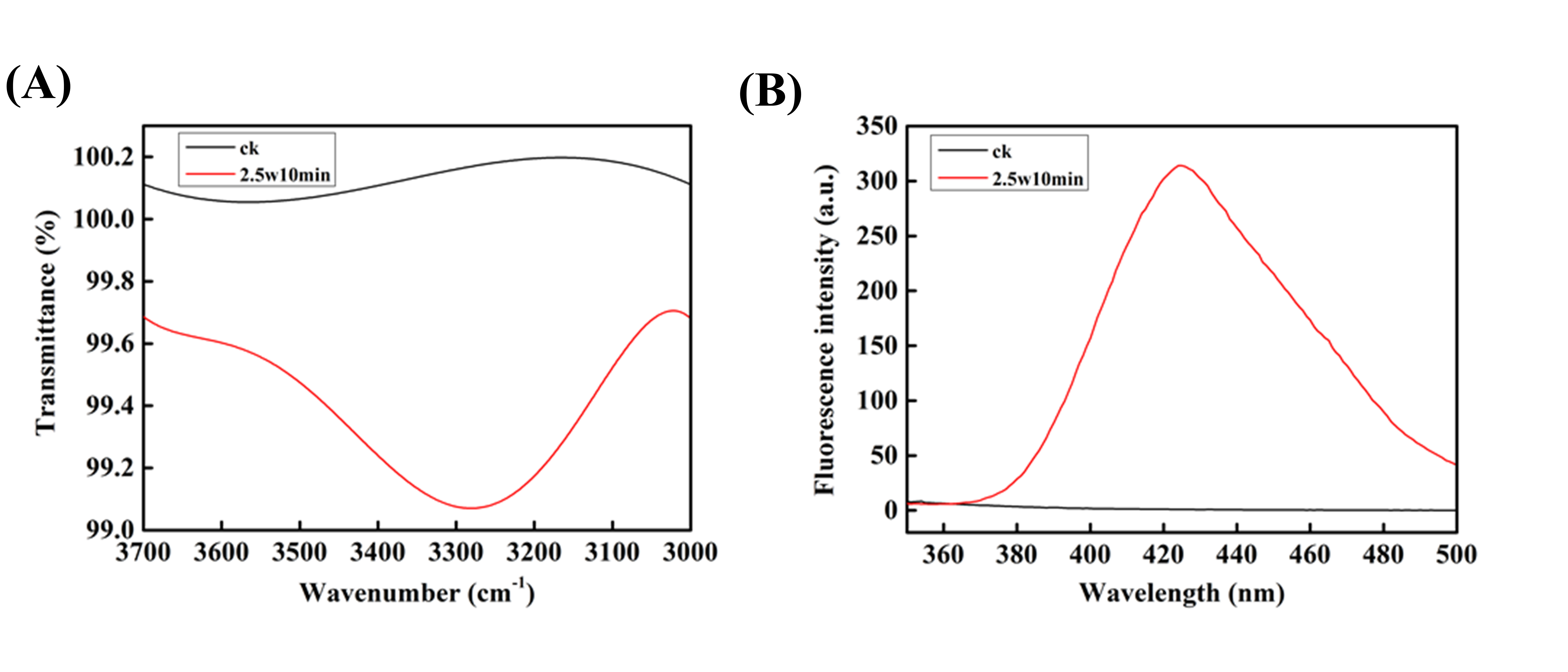

Supplement: Supplementary file 1 [file Table_1.doc]
